# The Utility of Carbohydrate-Deficient Transferrin (CDT) Test for Regranting Driver’s License After Repeat Drunk-Driving in Taiwan

**DOI:** 10.1192/j.eurpsy.2025.1516

**Published:** 2025-08-26

**Authors:** M.-H. Hsieh, R.-P. Lee, S.-C. Huang

**Affiliations:** 1 Psychiatry, Tungs’ Taichung MetroHarbor Hospital, Taichung City, Taiwan, Province of China

## Abstract

**Introduction:**

Carbohydrate-Deficient Transferrin (CDT), a biomarker for excessive alcohol consumption, has been used in driver’s license regranting programs in the UK and other European countries, but relevant data is lacking in Taiwan. This study collected %CDT data from repeat drunk driving offenders to monitor their alcohol consumption and aims to provide local data to inform policies in Taiwan.

**Objectives:**

The objective of this study is to assess whether alcohol consumption decreases over a 12-month period, as measured by %CDT values and Alcohol Use Disorders Identification Test - Consumption (AUDIT-C) scores.

**Methods:**

**Study Design**

This study was conducted from 2020 to 2023, involving 65 recidivist drunk drivers whose licenses had been revoked and who sought to reapply. Participants were referred to our hospital for a one-year, monthly evaluation and intervention program. Data were collected on gender, age, marital status, education level, employment status, years of alcohol consumption, primary type of alcohol consumed, DSM-5 severity, AUDIT, and AUDIT-C scores. Blood tests, including GGT, AST, ALT, triglycerides, cholesterol, and %CDT, along with AUDIT-C assessments, were conducted at the 1st, 4th, 7th, and 11th evaluations.

**Results:**

Basic demographic information is shown in Image 1. There was no significant difference in %CDT before and after the intervention (p = 0.332), with values of 1.99% and 2.28%, respectively. Among 42 participants who completed the 12-month intervention, %CDT showed no significant change (p = 0.46; 1.93% vs. 1.83%). For 23 participants who did not complete the intervention, %CDT also showed no significant difference (p = 0.219; 2.11% vs. 3.14%).

However, AUDIT-C scores significantly decreased across all groups. The total group’s scores dropped from 4.51 to 3.20 (p = 0.00091), the completion group from 4.00 to 2.60 (p = 0.011), and the non-completion group from 5.43 to 4.30 (p = 0.025). These results are shown in Image 2, and the correlations between baseline variables are displayed in Image 3.

**Image 1:**

**Figure:**
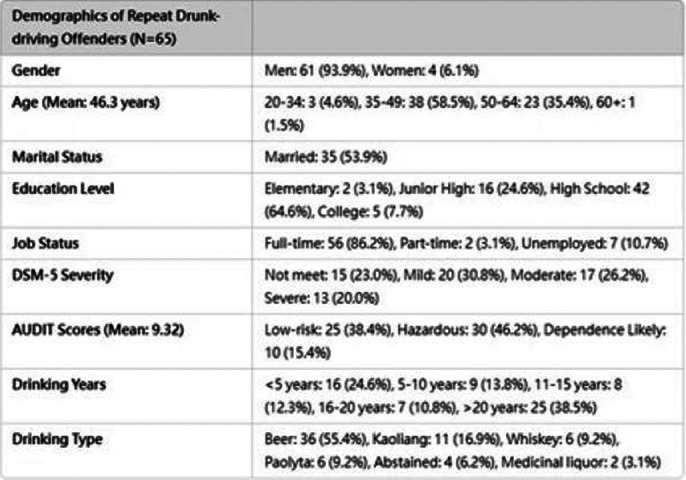

**Image 2:**

**Figure:**
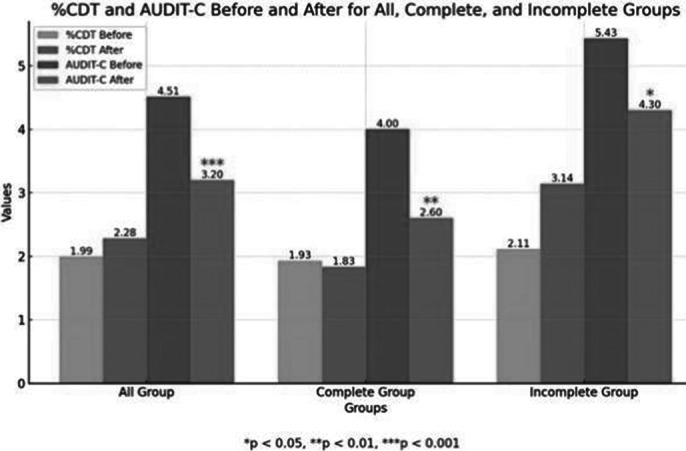

**Image 3:**

**Figure:**
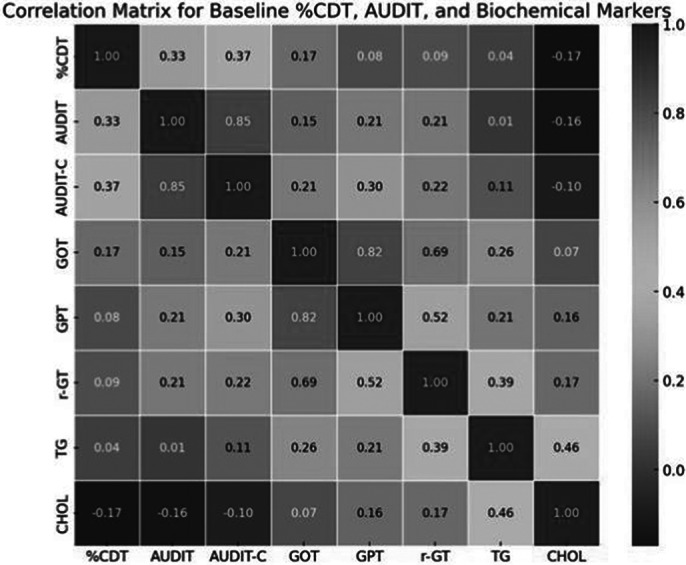

**Conclusions:**

Our study found that monitoring drunk-driving recidivists with %CDT every three months did not yield a statistically significant change in %CDT, but did result in a significant reduction in AUDIT-C scores. Fleming et al. (2004) suggest that psychological pressure from long-term biomarker monitoring may have a therapeutic effect, with bimonthly CDT follow-ups potentially beneficial. Thus, using %CDT for license reinstatement is feasible, though adjustments to assessment intervals and inclusion of other biomarkers should be considered.

Fleming et al., Alcohol Clin Exp Res 2004; 28: 1347–55.

**Disclosure of Interest:**

None Declared

